# C26:0-Carnitine Is a New Biomarker for X-Linked Adrenoleukodystrophy in Mice and Man

**DOI:** 10.1371/journal.pone.0154597

**Published:** 2016-04-28

**Authors:** Malu-Clair van de Beek, Inge M. E. Dijkstra, Henk van Lenthe, Rob Ofman, Dalia Goldhaber-Pasillas, Nicolas Schauer, Martin Schackmann, Joo-Yeon Engelen-Lee, Frédéric M. Vaz, Wim Kulik, Ronald J. A. Wanders, Marc Engelen, Stephan Kemp

**Affiliations:** 1 Laboratory Genetic Metabolic Diseases, Departments of Pediatrics and Clinical Chemistry, Academic Medical Center, University of Amsterdam, Amsterdam, The Netherlands; 2 Departments of Pediatrics and Pediatric Neurology, Emma Children’s Hospital, Academic Medical Center, University of Amsterdam, Amsterdam, The Netherlands; 3 Metabolomic Discoveries GmbH, Potsdam-Golm, Germany; 4 Department of Neurology, Academic Medical Center, University of Amsterdam, Amsterdam, The Netherlands; Huashan Hospital, Fudan University, CHINA

## Abstract

X-linked adrenoleukodystrophy (ALD), a progressive neurodegenerative disease, is caused by mutations in *ABCD1* and characterized by very-long-chain fatty acids (VLCFA) accumulation. Virtually all males develop progressive myelopathy (AMN). A subset of patients, however, develops a fatal cerebral demyelinating disease (cerebral ALD). Hematopoietic stem cell transplantation is curative for cerebral ALD provided the procedure is performed in an early stage of the disease. Unfortunately, this narrow therapeutic window is often missed. Therefore, an increasing number of newborn screening programs are including ALD. To identify new biomarkers for ALD, we developed an *Abcd1* knockout mouse with enhanced VLCFA synthesis either ubiquitous or restricted to oligodendrocytes. Biochemical analysis revealed VLCFA accumulation in different lipid classes and acylcarnitines. Both C26:0-lysoPC and C26:0-carnitine were highly elevated in brain, spinal cord, but also in bloodspots. We extended the analysis to patients and confirmed that C26:0-carnitine is also elevated in bloodspots from ALD patients. We anticipate that validation of C26:0-carnitine for the diagnosis of ALD in newborn bloodspots may lead to a faster inclusion of ALD in newborn screening programs in countries that already screen for other inborn errors of metabolism.

## Introduction

X-linked adrenoleukodystrophy (ALD) is a progressive neurodegenerative disorder caused by mutations in the *ABCD1* gene [1]. The disease is characterized by impaired degradation of very long-chain fatty acids (VLCFA; >C22) [2, 3], resulting in VLCFA accumulation in plasma and tissues [4]. ALD affects approximately 1 in 17.000 males [5] and has been diagnosed in all geographic regions and ethnic groups. There is no evidence that the prevalence varies with ethnic background [6]. Patients with ALD are asymptomatic at birth [7]. A study in neurologically asymptomatic young boys with ALD revealed that 80% had unrecognized adrenal insufficiency [8]. Virtually all male patients with ALD eventually develop progressive myelopathy (adrenomyeloneuropathy, AMN). The onset is typically between 20–30 years of age [7, 9]. This myelopathy progresses gradually, eventually causing severe disability. Recently, we showed that women with ALD are not merely carriers, but that >80% also develop signs of myelopathy [10]. A subset of male patients, however, develops a fatal cerebral demyelinating disease (cerebral ALD). The age of onset of cerebral ALD cannot be predicted. A newborn male patient has a 35–40% risk to develop cerebral ALD between the ages of 3 and 18 years, but cerebral ALD can also occur in adulthood [7, 9, 11]. Allogeneic hematopoietic stem cell transplantation (HSCT) is curative for cerebral ALD provided the procedure is performed in an early stage of the disease before extensive MRI white matter abnormalities are present [12]. Unfortunately, this therapeutic window is often missed.

If newborn screening for ALD is implemented cerebral ALD can be identified and treated in an early stage, thus substantially increasing the likelihood of a better outcome. This is one of the reasons why ALD is being added to an increasing number of newborn screening programs. For example, New York State initiated ALD newborn screening in 2014 and in Europe the Netherlands will start ALD newborn screening in the near future. In August 2015, the United States advisory committee on heritable disorders in newborns and children recommended ALD to be added to the recommended uniform screening panel (RUSP). Currently ALD newborn screening involves quantification of 1-hexacosanoyl-2-lyso-sn-3-glycero-phosphorylcholine (C26:0-lysoPC) by liquid chromatography-tandem mass spectrometry (LC-MS/MS) in dried blood spots [13]. While the method shows high sensitivity and specificity, the need for liquid chromatography to separate the different lysoPC species hampers high throughput screening. The procedure has been slightly modified [14, 15] to combine the existing method for acylcarnitine analysis and C26:0-lysoPC, which enables simultaneous extraction and screening for peroxisomal disorders, mitochondrial fatty acid oxidation disorders and organic acidurias. Recently, a method was developed that enables the quantification of amino acids, acylcarnitines, succinylacetone, and C26:0-lysoPC in a single dried bloodspot punch [16].

The neurometabolic consequences of ALD protein (ALDP) deficiency and the subsequent increase in VLCFA levels have not yet been resolved at the cellular level. The absence of a ALD mouse model that mimics the disease in humans has been a major limitation in unraveling the mechanism underlying the pathogenesis of ALD. The *Abcd1*^*y/-*^ knockout mouse develops a mild phenotype that resembles the myelopathy of ALD, but only at around 20 months of age [17]. There is indirect evidence that an increase in VLCFA levels in *Abcd1* knockout mice may result in an earlier onset of disease. For example, *Abcd1* knockout mice that were put on a high-fat diet showed structural abnormalities in the adrenal gland on electron microscopy [18]. In readily accessible material from patients, such as plasma, fibroblasts, or blood cells, total C26:0 levels do not correlate with phenotype [19]. However, biochemical analysis of normal-appearing grey and white matter that was dissected from frontal, parietal or occipital lobes from 17 ALD patients and 19 age-matched controls revealed a correlation between VLCFA levels and clinical phenotype [20]. Support for a toxic effect of VLCFA came from the demonstration that C24:0-lysoPC injection into wild type mouse brain resulted in widespread microglial activation and apoptosis [21]. Importantly, C16:0-lysoPC injections did not cause these effects. Studies using rat neural cells revealed that exposure of oligodendrocytes and astrocytes to VLCFA, but not C16:0, caused cell death [22]. Challenging neural cells with VLCFA induced depolarization of mitochondria *in situ* and caused deregulation of intracellular calcium homeostasis [22]. Long before the first neuropathological lesions are detectable in the spinal cord of *Abcd1* knockout mice there is oxidative damage to proteins [23].

With the aim to generate an ALD mouse with increased VLCFA levels in the central nervous system, we developed an *Abcd1* knockout mouse with a Cre-inducible *ELOVL1* transgene: the key enzyme in VLCFA synthesis [24, 25]. Based on the above studies we anticipated that increased VLCFA levels in the central nervous system could impact the clinical phenotype of the *Abcd1* knockout mouse and may result in the identification of new biomarkers.

## Materials and Methods

### Patient and Control Samples

Samples from ALD patients were available from a previous study that was approved by the Institutional Review Board (Medisch Ethische Toetsings Commissie) of the Academic Medical Center (BEZA trial [26]). Written informed consent was received from each patient. Samples for control values were obtained from the Laboratory Genetic Metabolic Diseases from anonymized samples from individuals who were examined in our institute for therapeutic control or exclusion of inherited metabolic disease. All samples were collected according to the institutional guidelines for sampling.

### Ethics Statement and Animal Care

Mice were bred in the animal housing facility of the AMC, had ad libitum access to water and standard rodent food, and were kept on a 12 hour light and dark cycle. For the care and use of animals utilized in this research, we monitored the animals twice per week. None of animals showed severe illness or died during the experiments. A protocol for early euthanasia/humane endpoints is performed if one of the following criteria is met: the loss of body weight more than 15% or a wound that cannot be improved after medication. For tissue collection, the mice were sacrificed by introduction of 100% carbon dioxide into a bedding-free cage with the lid closed at a rate sufficient to induce rapid anesthesia, with death occurring within 2.5 minutes. All animal experiments were approved by the institutional review board for animal experiments at the Academic Medical Center, University of Amsterdam (Amsterdam, The Netherlands).

### Generation of *ELOVL1* Knock-In Mice

**Fig 1:**
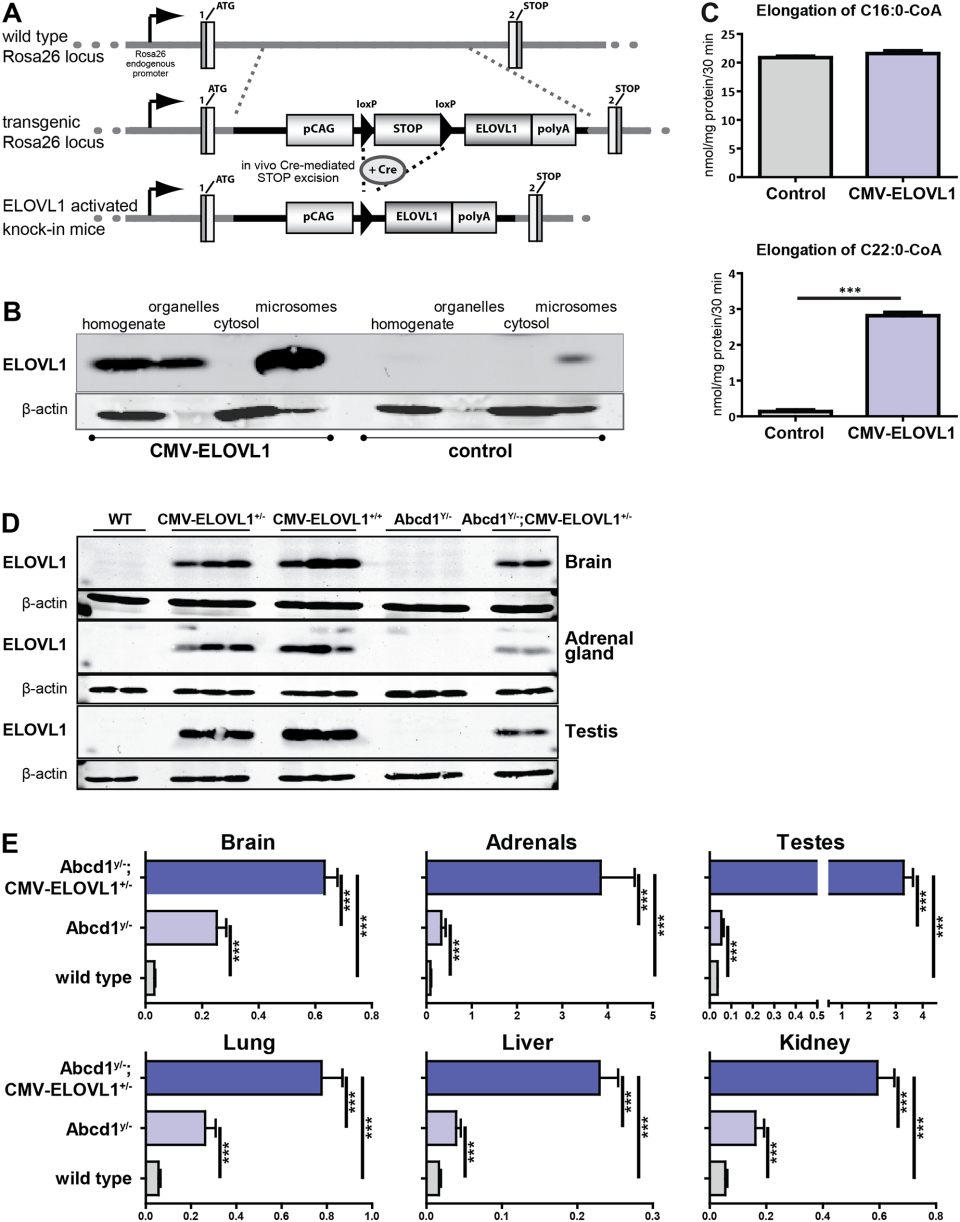
Generation and characterization of *Abcd1*;*CMV-ELOVL1* mice. **(A)** Schematic presentation of the Rosa26^CAG-STOPflox/flox-ELOVL1TG^ transgenic construct (black line). The grey line indicates the wild type *Rosa26* gene. *ELOVL1* transgene expression is prevented by the upstream floxed STOP element. The *ELOVL1* transgenic construct consists of a loxP flanked STOP cassette positioned between the human *ELOVL1* coding cDNA and the upstream ubiquitous CAG promoter. Crossing the transgenic mice with a Cre-driver strain activates *ELOVL1* transgene expression. **(B)** ELOVL1 protein expression in total homogenates, organelle and cytosolic fractions and purified microsomes derived from livers from wild type mice (control) and wild type mice heterozygous (*CMV-ELOVL1*^*+/-*^) for the activated *ELOVL1* transgene. **(C)** C16:0-CoA and C22:0-CoA elongation capacity in mouse liver microsomes (n = 5) derived from wild type mice (control) and wild types heterozygous (*CMV-ELOVL1*^*+/-*^) for the activated *ELOVL1* transgene. **(D)** ELOVL1 protein expression in brain, adrenal gland and testis from wild type (WT), wild type heterozygous (*CMV-ELOVL1*^*+/-*^) or homozygous (*CMV-ELOVL1*^*+/+*^) for the activated *ELOVL1* transgene, *Abcd1*^*y/-*^ knockout and *Abcd1*^*y/-*^ knockout heterozygous for the activated *ELOVL1* transgene (*Abcd1*^*y/*-^;*CMV-ELOVL1*^*+/-*^) mice. **(E)** C26:0 levels (in nmol/mg protein) in tissues derived from wild type (n = 6), *Abcd1*^*y/-*^ knockout (n = 6) and *Abcd1*^*y/*-^;*CMV-ELOVL1*^*+/-*^ (n = 2) mice. Data are mean ± SD. ***p<0.001 by unpaired student’s t-test.

The *ELOVL1* transgenic construct and mouse model were commercially generated by GenOway (Lyon, France). GenOway’s validated *Rosa26* “Quick Knock-in^™^” approach was used to introduce a single copy of the human *ELOVL1* transgenic cassette into the *Rosa26* locus on chromosome 6 through homologous recombination in embryonic stem cells. Briefly, the human *ELOVL1* cDNA fragment was subcloned into a GenOway’s G136 plasmid containing the human growth factor polyA cassette. Then the *ELOVL1*-polyA cassette was subcloned into GenOway’s G212 vector downstream of the pCAG promoter and the transcriptional STOP cassette flanked by two *lox*P sites. The linearized construct (Fig 1) was transfected into mouse 129SV ES cells according to GenOway's standard electroporation procedures (i.e. 5×10^6^ ES cells in presence of 40 μg of linearized plasmid, 260 volt, 500 μF). Positive selection was started 48 hours after electroporation by addition of 200 μg/ml of G418. Approximately 223 G418-resistant clones were screened for the correct homologous recombination event at the *Rosa26* locus by PCR and southern blotting. Two correctly recombined ES clones were used for injection into C57BL/6J blastocysts. Injected blastocysts were re-implanted into OF1 pseudo-pregnant females and allowed to develop to term. A total of 9 male chimeric mice were produced from the 2 injection sessions. Four highly chimeric males (displaying 80% chimerism) were each mated with 2 C57BL/6J Flp-deleter females to allow germline excision of the neomycin selection cassette. Agouti F1 progeny were screened for assessing the Flp-mediated excision event of the neomycin cassette on the *ELOVL1* allele by PCR and southern blotting, using genomic DNA isolated from tail biopsies. As there was mosaicism for the Flp *ELOVL1* allele in F1 mice, these mice were then crossed with wild-type C57BL/6J mice to generate a pure line of Flp excised heterozygous *ELOVL1* knock-in mice. PCR and southern blot screening confirmed the successful generation of 3 males and 2 females heterozygous for the *Rosa26* neomycin-excised *ELOVL1* knock-in allele. These mice are referred to as *Rosa26*^CAG-STOPflox/flox-ELOVL1TG^.

### Generation of *CMV*-*ELOVL1* Mice

The C57BL/6J *ELOVL1* conditional knock-in strain (*Rosa26*^CAG-STOPflox/flox-ELOVL1TG^) was crossed with a CMV-Cre driver strain (B6.C-Tg(CMV-cre)1Cgn/J, The Jackson laboratory, Bar Harbor, ME, USA) to obtain mice overexpressing ELOVL1 in all tissues (referred to as *CMV*-*ELOVL1*)

### Generation of *Abcd1*-*CMV*-*ELOVL1* Mice

The C57BL6/J *CMV-ELOVL1* strain was crossed with our in-house in-bred Swiss *Abcd1* knockout strain (defined as 10 generations back-crossed to Swiss, followed by >20 generations of brother-sister crossings) to obtain *Abcd1* knockout mice ubiquitously overexpressing ELOVL1 on a mixed Swiss, C57BL/6J background (referred to as *Abcd1*;*CMV*-*ELOVL1*).

### Generation of *Abcd1*-*Cnp*-*ELOVL1* Mice

The C57BL/6J *ELOVL1* conditional knock-in strain (*Rosa26*^CAG-STOPflox/flox-ELOVL1TG^) was crossed with the C57BL/6J *Abcd1* knockout strain ([27] a kind gift from K.A. Nave, Max Planck Institute for Experimental Medicine, Göttingen, Germany)) to obtain an *Abcd1* knockout–*ELOVL1* conditional knock-in strain (*Abcd1*-*Rosa26*^CAG-STOPflox/flox-ELOVL1TG^). Subsequently, these mice were crossed with a Cnp-Cre driver strain ([28] C57BL/6J-Cnp-Cre, a kind gift from K.A. Nave, Max Planck Institute for Experimental Medicine, Göttingen, Germany) to obtain *Abcd1* knockout mice overexpressing ELOVL1 in oligodendrocytes on a pure C57BL/6J background (referred to as *Abcd1*;*Cnp*-*ELOVL1*). Mice were housed at 21 ± 1°C, 40–50% humidity, on a 12 h light-dark cycle, with *at libitum* access to water and a standard rodent diet.

### Mouse Genotyping

Genomic DNA was isolated from toe clippings derived from mice 6 days of age using the Phire Animal Tissue direct PCR kit (Thermo Scientific, Waltham, MA, USA) according to the manufacturer’s procedure. For the PCR, only the primer sequences and critical PCR steps are provided: *Abcd1* genotyping was done in a multiplex PCR with Abcd1-WT-F, 5’- CACAGCCTCTCTCCTTAAGACC -3’; Abcd1-WT-R, 5’- CTCGTTGTCTAGGCAACTGG -3’ and Abcd1-Neo-R, 5’- CTTCTATCGCCTTCTTGACG -3’ (Mg^2+^ was 2.0 mM final concentration, touch down PCR (initial annealing at 64°C reduced to 58°C with 0.5°C steps/cycle, the wild type amplicon is 217 bp and the *Abcd1* knockout amplicon is 117 bp). ELOVL1 knock-in genotyping was done with Rosa26-F, 5’- CAATACCTTTCTGGGAGTTCTCTGC-3’, and Rosa26-R, 5’- CTGCATAAAACCCCAGATGACTACC-3’ for detection of the *Rosa26* wild type allele and ELOVL1-F, 5’- GAAAAAGCACATGACAGCCATTCAGC-3’, and ELOVL1-R, 5’- CCCTACAGGTTGTCTTCCCAACTTGC-3’ for detection of the Neo-excised recombined *Rosa26* locus (Mg^2+^ was 1.5 mM final concentration, annealing temperature was 65°C, the *Rosa26* amplicon is 304 bp and the *ELOVL1* amplicon is 485 bp). CMV-Cre genotyping was done with Cre-F, 5’- GGGATTGCTTATAACACCCTGTTACG-3’, and Cre-R, 5’- TATTCGGATCATCAGCTACACCAGAG-3’ (Mg^2+^ was 1.5 mM final concentration, annealing temperature was 55°C, the Cre amplicon is 213 bp). Cnp-Cre genotyping was done in a multiplex PCR with CNP-F, 5’- GCCTTCAAACTGTCCATCTC -3’, CNP-R, 5’- CCCAGCCCTTTTATTACCAC -3’, and Puro3, 5’- CATAGCCTGAAGAACGAGA -3’ (Mg^2+^ was 1.5 mM final concentration, annealing temperature was 55°C, the wild type amplicon is 643 bp and the Cnp-Cre amplicon is 357 bp).

### Purification of Mouse Liver Microsomes

Microsomes were isolated from livers from wild type and ubiquitous *ELOVL1* over-expressing *Abcd1* knockout mice (*Abcd1*^*y/-*^*;CMV-ELOVL1*^+/-^) by differential centrifugation. Livers were washed with ice-cold homogenization buffer containing 250 mM sucrose, 2 mM EDTA, 2 mM DTT and 5 mM MOPS (pH 7.4), minced and homogenized with 20 strokes of a dounce homogenizer while kept on ice. A post-nuclear supernatant was produced by centrifugation at 600 g for 10 min. The supernatant was centrifuged at 22,500 g for 10 min and the pellet was discarded. To obtain a microsomal fraction, the supernatant was centrifuged for 1 h at 100,000 g. To remove any residual fatty acids the pellet was resuspended in homogenization buffer containing 10 mg/mL methyl-β-cyclodextrin and sonicated four times for 5 seconds at 7W. The microsomal membranes were collected by centrifugation at 100,000 g for 1 h. Finally, the microsomes were resuspended in homogenization buffer and stored at -80°C in 100 μL aliquots until further use. All steps were carried out at 4°C. Protein concentration was determined using Pierce^®^ BCA protein assay (Thermo Scientific, Waltham, MA, USA) with human serum albumin as standard.

### Fatty Acid Elongation Assay

The reaction mixture contained 50 mM potassium phosphate buffer (pH 6.5), 10 mg/mL α-cyclodextrin, 1 mM NADPH, 5 μM rotenone, 60 μM [2-^14^C] malonyl-CoA (6.5 dpm/pmol) (American Radiolabeled Chemicals, St. Louis, MO, USA) and 20 μM C16:0-CoA or 20 μM C22:0-CoA (Avanti Polar Lipids, Alabaster, AL, USA), 1 mM NADPH and 10 mg/ml α-cyclodextrin in a total volume of 200 μL. The reaction mixture was pre-incubated for 2 min at 37°C and started by the addition of 100 μg microsomal protein. After 30 min at 37°C the reaction was stopped by adding 200 μL 5 M KOH in 10% methanol and saponified at 65°C for 1 h. After acidification, by adding 200 μL 5 N HCl and 200 μL 96% ethanol, fatty acids were extracted three times with 1 mL hexane and the hexane phases were collected in a scintillation vial. To each scintillation vial 10 mL Ultima-Gold scintillation cocktail (Perkin Elmer, Waltham, MA, USA) was added and the radioactivity was counted.

### Protein Blot Analysis

The polyclonal antibody against the C-terminal amino acids of human ELOVL1 (LQQNGAPGIAKVKAN) was generated by Eurogentec (Eurogentec, Liege, Belgium). Lysates of tissues from 20 week old wild type, *Abcd1* knockout, *Abcd1*^*-/y*^*;CMV-ELOVL1* and *Abcd1*^*y/-*^*;Cnp-ELOVL1* (n = 3 to 5 per genotype) were prepared by sonication in PBS containing protease inhibitor cocktail (Roche, Indianapolis, IN, USA). After sonication, samples were diluted 1:1 in sample buffer containing 8 M urea. Protein samples (70 μg) were separated on 12.5% SDS-PAGE gels and transferred onto nitrocellulose membranes. Membranes were blocked with 2% BSA in PBS containing 0.1% Tween20 (w/v) and probed the primary polyclonal antibody against human ELOVL1 at 1:10 dilution in blocking buffer [24]. Goat anti-rabbit IgG IRDye 800CW (1:10.000, LICOR Biosciences, Lincoln, NE, USA) was used as a secondary antibody. Visualization of the signal was done with the Odyssey IR imaging system (LI-COR Biosciences, Lincoln, NE, USA).

### Non-Targeted Metabolic Profiling

Bloodspots were generated by collecting 50 μL blood from the vena saphena from C57BL6/6 and Swiss mice. All subsequent steps were carried out at Metabolomic Discoveries GmbH (Potsdam, Germany; www.metabolomicdiscoveries.com) essentially as described [29], but with improvements. Discs of 6 mm diameter were extracted with 1 ml 80% (v/v) methanol and sonicated for 1 h at 4°C. 100 μl was used for HPLC-TOF-MS analyses. LC separation was performed using a SeQuant ZIC HILIC column (Merck) operated by an Agilent 1290 UPLC system (Agilent, Santa Clara, CA, USA). The LC mobile phase was A) 10 mM ammonium acetate in 95% (v/v) acetonitrile and 5% (v/v) water B) 10 mM ammonium acetate in 95% (v/v) water with a gradient from 0% B to 90% over 5 min, to 95% at 6.5 min and 100% at 8 min, subsequently equilibrate. The flow rate was 400 μl/min, injection volume 1 μl. Mass spectrometry was performed using a high-resolution 6540 QTOF/MS Detector (Agilent, Santa Clara, CA, USA) operated in positive and negative ionization modes with a mass accuracy of <2ppm. For UPLC-QTOF/MS, metabolites were identified or putatively annotated in comparison to Metabolomic Discoveries' database entries of authentic standards and IDEOM database entries through peak mass within 5 ppm mass accuracy and retention time.

### Total Fatty Acid Analysis

Fatty acids were analyzed by electrospray ionization mass spectrometry (ESI-MS) as described previously [30].

### LysoPC and Acylcarnitine: Preparation of Standards and Calibrators

Methanol, acetonitrile and formic acid were of analytical grade. C20:0-lysoPC, C26:0-lysoPC and ^2^H_4_-C26:0-lysoPC were purchased from Avanti Polar Lipids (Alabaster, AL, USA). C26:0-carnitine was purchased from Dr. H.J. ten Brink (VU Medical Center, Amsterdam). ^2^H_4_-C26:0-carnitine as internal standard was synthesized in house from 12,12,13,13-^2^H_4_-C26:0 (CDN Isotopes, Pointe-Claire, Quebec Canada), thionylchloride (Merck, Kenilworth, NJ, USA) and L-carnitine (Sigma-Aldrich, St. Louis, MO, USA), essentially as described [31]. Stock- and standard solutions were prepared in methanol. Internal standard solutions of 1 μmol/L ^2^H_4_-C26:0-carnitine and 1 μmol/L ^2^H_4_-C26:0-lysoPC in methanol were used for sample preparation. Standard solutions of 0.1 μmol/L C26:0-carnitine, 0.1 μmol/L C20:0-lysoPC and 0.1 μmol/L C26:0-lysoPC in methanol were used for calibration.

### LysoPC and Acylcarnitine: Sample Preparation

A combined punch of a dried bloodspot (¼ inch in diameter) and 10 μL of internal standard were extracted with 0.5 mL of methanol by ultrasonication for 5 min. The extract was dried (N_2_, 40°C) and reconstituted in 50 μL of methanol. 10 μL was used for analysis.

For mouse tissues, 0.2 mg and 10 μL of internal standard were extracted with 0.5 mL of acetonitrile by ultrasonication for 5 min. After full speed centrifugation (5 min, 4°C) the supernatant was dried (N_2_, 40°C) and reconstituted in 50 μL of methanol. 10 μL was used for analysis.

### UPLC-MS/MS

The ACQUITY UPLC system (Waters, Milford, MA, USA) consisted of a binary solvent manager, a vacuum degasser, a column heater and sample manager. The column temperature was maintained at 50°C. The samples were injected onto a Kinetex C8 column, 50 × 2.1 mm, 2.6 μm particle diameter (Phenomenex, Torrance, CA, USA). The acylcarnitines were separated by a linear gradient between solution A (0.1% formic acid in H_2_O) and solution B (0.1% formic acid in methanol). The gradient was as follows: at T = 0 min: 36% A, 64% B, flow 0.4 mL/min towards T = 6 min: 0% A, 100% B, flow 0.4 mL/min; T = 6–11 min: 0% A, 100% B, flow 0.4 mL/min, and T = 11–11.1 back to 36% A, 64% B, flow 0.4 mL/min. A Quattro Premier XE (Waters, Milford, MA, USA) was used in the positive electrospray ionization mode. Nitrogen was used as desolvation gas (900 L/h) and cone gas (50 L/h). Desolvation temperature was 350°C, capillary voltage was 3.5 kV and the source temperature was 130°C. Argon was used as collision gas (2.5 x 10e-3 mbar). For the very long-chain acylcarnitines and lysophosphatidylcholines (lysoPC) multiple reaction monitoring (MRM) traces were acquired with optimized cone voltage and collision energy for each transition with a dwell time of 0.01 s (Table 1).

**Table 1:** MRM, cone voltage and collision energy for each acylcarnitine and lysoPC.

Metabolite	MRM	Cone voltage (V)	Collision energy (eV)
**C22:0-carnitine**	484.40 > 85.00	49	30
**C24:0-carnitine**	512.50 > 85.00	51	31
**C26:0-carnitine**	540.50 > 85.00	54	32
^**2**^**H**_**4**_**-C26:0-carnitine**	544.50 > 85.00	54	32
**C22:0-lysoPC**	580.40 > 104.10	47	28
**C24:0-lysoPC**	608.50 > 104.10	50	29
**C26:0-lysoPC**	636.50 > 104.10	53	31
^**2**^**H**_**4**_**-C26:0-lysoPC**	640.50 > 104.10	53	31

### Data Analysis

Data are expressed as means ± SD of measurements. Statistical comparisons were performed using the unpaired student’s test, and significance was defined as: * = P < 0.05, ** = P < 0.01 and *** = P < 0.001.

## Results and Discussion

### Generation of *ELOVL1* Transgenic Mice

ELOVL1 is the essential enzyme in the elongation of C22:0 to C26:0 [24, 25, 32]. To investigate the *in vivo* effect of ELOVL1 overexpression on VLCFA homeostasis, we generated a Cre-inducible *ELOVL1* transgenic mouse (*Rosa26*^*CAG*-STOPflox/flox-ELOVL1TG^). The *ELOVL1* transgene consists of a loxP flanked STOP element positioned between the *ELOVL1* coding DNA sequence and its upstream chicken β-actin/CMV immediate early enhancer fusion promoter (Fig 1A).

As a proof of concept that enhanced ELOVL1 expression results in increased ELOVL1 protein levels and increased VLCFA levels, homozygous *Rosa26*^*CAG*-STOPflox/flox-ELOVL1TG^ mice were crossed with a Cre-driver strain that expresses Cre recombinase under the control of a human cytomegalovirus (CMV) promoter (CMV-Cre^+/-^). F1 mice were either heterozygous for the activated *ELOVL1* transgene (*CMV*-*ELOVL1*^+/-^) in all tissues or negative for the activated *ELOVL1* transgene (controls). The efficacy of transgene activation was assessed in mouse liver homogenates generated from *CMV-ELOVL1*^*+/-*^ and control mice. An organelle fraction was prepared from which microsomes were isolated.

Protein blot analysis revealed a strong increase in ELOVL1 protein in livers derived from *CMV-ELOVL1*^*+/-*^ mice, but not in controls (Fig 1B). ELOVL1 was present exclusively in the organelle fraction, with a strong expression in the microsomes, indicating correct targeting to the endoplasmic reticulum [33]. While only a very faint ELOVL1 signal was detectable in the microsomal fraction of the control mice, a strong ELOVL1 signal was present in *CMV-ELOVL1*^*+/-*^ mice (ratio 1:20). In control mice ELOVL1 was only detectable in purified microsomes. Taken together, these data show that activation of the *ELOVL1* transgene results in a marked increase in ELOVL1 expression and that the protein is localized correctly to the ER membrane. Microsomes from control and *CMV-ELOVL1*^*+/-*^ mice were used to determine the fatty acid elongation capacity for both long-chain fatty acids (C16:0-CoA) and VLCFA (C22:0-CoA) (Fig 1C). No difference between control and *CMV-ELOVL1*^*+/-*^ mice was seen when microsomes were incubated with C16:0-CoA. This was expected as C16:0-CoA is a substrate for the long-chain fatty acyl elongase, ELOVL6 [24, 34]. In contrast, microsomes from *CMV-ELOVL1*^*+/-*^ mice had a 25-fold higher elongation activity for C22:0-CoA, which is in agreement with the substrate specificity of ELOVL1 [24, 32].

To assess the biochemical effect of ELOVL1 over-expression in *Abcd1* knockout mice, the *CMV-ELOVL1* line was crossed with *Abcd1* knockouts. ELOVL1 protein and VLCFA levels were measured in wild type, *Abcd1*^*y/-*^ knockout and *Abcd1*^*y/-*^*;CMV-ELOVL1*^*+/-*^ mice. Protein blot analysis of brain, adrenal gland and testis demonstrated a strong expression of transgenic ELOVL1 (Fig 1D).

Total C26:0 levels were measured in tissues from 4 month old wild type, *Abcd1*^*y/-*^ knockout and *Abcd1*^*y/-*^*;CMV-ELOVL1*^*+/-*^ mice. Compared to wild type mice, in *Abcd1*^*y/-*^ knockouts C26:0 levels were increased 8-fold in brain, 4-fold in adrenals, 1.5-fold in testes, 4.5-fold in lung, 2.5-fold in liver, and 3.5-fold in kidney (Fig 1E). In *Abcd1*^*y/-*^*;CMV-ELOVL1*^*+/-*^ mice C26:0 levels were increased 20-fold in brain, 44-fold in adrenals, 90-fold in testes, 14-fold in lung, 14-fold in liver and 11-fold in kidney (Fig 1E). Compared to *Abcd1*^*y/-*^ knockout mice, C26:0 levels in *Abcd1*^*y/-*^*;CMV-ELOVL1*^*+/-*^ mice were further increased by 2.5-fold in brain, 12-fold in adrenal, 60-fold in testis, 3-fold in lung, 6-fold in liver and 4-fold in kidney (Fig 1E). These data demonstrate that *in vivo* overexpression of ELOVL1 results in a strong increase in C26:0 levels.

### Possibly Early Lethal Phenotype in *Abcd1*;*CMV-ELOVL1* Mice

**Fig 2:**
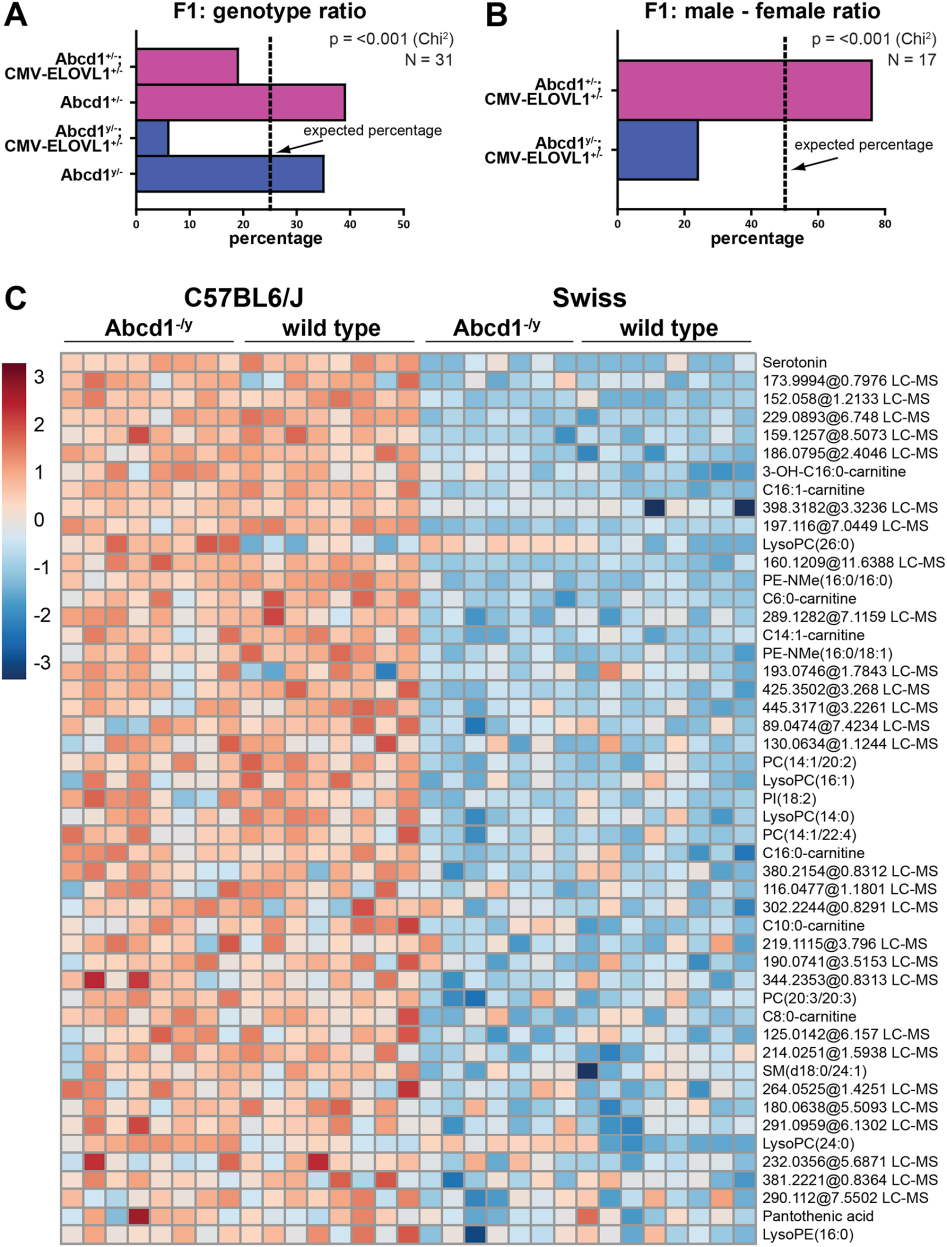
Possibly early lethal phenotype in *Abcd1*;*CMV-ELOVL1* mice. **(A)** Distribution of the actual F1 genotypes of 4 independent crosses between *Abcd1* knockout females (*Abcd1*^*-/-*^) with males heterozygous for the activated *ELOVL1* transgene (*CMV-ELOVL1*^*+/-*^). Males are in blue and females in pink. The dashed lines indicate the expected percentages. **(B)** The actual male to female ratio in the F1 generation of 4 independent crosses between *Abcd1* knockout females (*Abcd1*^*-/-*^) with males homozygous for the activated *ELOVL1* transgene (*CMV-ELOVL1*^*+/+*^). **(C)** Untargeted metabolomics using bloodspots derived from Swiss and C57BL6/J wild type and *Abcd1* knockout mice reveals strong differences in the metabolome between both background strains. The color in the heat map reflects the global metabolite abundance level according to the z-score. The 50 most significant different metabolites are shown (*P* < 0.05) as determined by a Welch’s t-test.

Breeding results with the *Abcd1;CMV-ELOVL1* line provided potential evidence for the early phenotype in *Abcd1*^*y/-*^*;CMV-ELOVL1*^*+/-*^ male mice. Homozygous *Abcd1* knockout females (*Abcd1*^*-/-*^) were crossed with males heterozygous for the activated *ELOVL1* transgene (*CMV-ELOVL1*^*+/-*^). In the F1, an equal genotype distribution was expected (25% for each genotype) with females being either *Abcd1*^*+/-*^ or *Abcd1*^*+/-*^*;CMV-ELOVL1*^*+/-*^ and males being either *Abcd1*^*y/-*^ or *Abcd1*^*y/-*^*;CMV-ELOVL1*^*+/-*^. Genotype analysis of 31 pups from 4 independent breeding pairs, however, revealed a significant underrepresentation in the number of *Abcd1*^*y/-*^*;CMV-ELOVL1*^*+/-*^ males. The expected and actual genotype-distribution is presented in Fig 2A. Although 25% of the newborn mice were expected to be *Abcd1*^*y/-*^*;CMV-ELOVL1*^*+/-*^ males, only 6% males with this genotype were born (Chi^2^ *P* = < 0.001). Additional evidence for a possibly early phenotype came from a second breeding strategy. Homozygous *Abcd1*^*-/-*^ knockout females were crossed with male mice homozygous for the activated *ELOVL1* transgene (*CMV-ELOVL1*^*+/+*^). The F1 expected genotypes were *Abcd1*^*+/-*^*;CMV-ELOVL1*^*+/-*^ (females) and *Abcd1*^*y/-*^*;CMV-ELOVL1*^*+/-*^ (males) with an equal female to male ratio. Genotyping analysis of 17 pups from 4 independent mating pairs, however, revealed an actual female to male ratio of 3:1 (Chi^2^ *P* = < 0.001) (Fig 2B). Taken together, these data indicate the onset of a possibly early lethal phenotype.

There are at least three possible explanations for these observations, namely: 1) compared to normal sperm cells, ELOVL1 over-expressing sperm cells may be affected in their motility, which could affect successful fertilization, 2) *Abcd1*^*y/-*^*;CMV-ELOVL1*^*+/-*^ males may already die in the prenatal stage, or 3) *Abcd1*^*y/-*^*;CMV-ELOVL1*^*+/-*^ pups may die immediately after birth. Alternatively, we cannot rule out the possibility that the mixed C57BL6/Swiss background of the *Abcd1;CMV-ELOVL1* line affects the early phenotype. This is nicely illustrated in Fig 2C that shows the results of an untargeted comprehensive metabolite profiling of bloodspots derived from C57BL6/J and Swiss wild type and *Abcd1* knockouts. We observed strong differences in the metabolome between the two mouse strains. The untargeted metabolomics data set was used for hierarchical clustering using MetaboAnalyst (http://metaboanalyst.ca/). In one dimension, this analysis perfectly clustered the four different groups of mice according to the background strain, i.e. Swiss or C57BL6/J (Fig 2C). This highest difference between Swiss and C57BL6/J mice was observed for serotonin. Interestingly, clustering revealed specific differences between Swiss and C57BL6/J mice in different lipid species and acylcarnitines. Both in Swiss and C57BL6/J *Abcd1* knockouts C26:0-lysoPC and to a lesser extend C24:0-lysoPC were highly elevated when compared to wild types.

However, because tissue VLCFA levels were uniformly elevated in a manner that does not reflect the biochemical signature of ALD (Fig 1E), we determined that the *Abcd1;CMV-ELOVL1* model had limited utility, and decided not to pursue studies with this line any further.

### Targeting ELOVL1 Expression to Oligodendrocytes

Whereas normal CNS myelin contains mostly long-chain fatty acids (C16 to C20), myelin in ALD patients contains large amounts of VLCFA [7, 35]. To generate *Abcd1* knockout mice with increased VLCFA levels in the central nervous system on the C57BL6/J background, we crossed three parental strains, i.e. C57BL6/J *Abcd1*^*y/-*^, C57BL6/J *Rosa26*^*CAG-STOPflox/flox-ELOVL1TG*^, and C57BL6/J *Cnp*^*tm1(cre)Kan*^, which expresses Cre recombinase under the control of the endogenous 2’,3’-cyclic nucleotide phosphodiesterase gene (*Cnp*) promoter [28]. In brain development, Cnp is active in oligodendrocytes [28]. The *Abcd1* knockout mice with ELOVL1 overexpression restricted to oligodendrocytes are referred to as *Abcd1;Cnp-ELOVL1*.

To generate Abcd1^y/-^;Cnp-ELOVL1^+/-^ males the following breeding scheme was used: Abcd1^-/-^;Cnp-ELOVL1^+/-^ females were crossed with Abcd1^y/-^;ELOVL1^-/-^ (ELOVL1 transgenic, but not active due to the absence of Cre) males. The F1 generation should consist of an equal genotype distribution with males being either Abcd1^y/-^;ELOVL1^-/-^ or Abcd1^y/-^;Cnp-ELOVL1^+/-^ and females being either Abcd1^-/-^; ELOVL1^-/-^ or Abcd1^-/-^;Cnp-ELOVL1^+/-^. Genotyping results of 80 mice from 14 litters revealed a normal male to female ratio (46% versus 54%). The actual Mendelian ratio for all genotypes was 20% Abcd1^y/-^;ELOVL1^-/-^, 34% Abcd1^y/-^;Cnp-ELOVL1^+/-^, 28% Abcd1^-/-^; ELOVL1^-/-^, and 19% Abcd1^-/-^;Cnp-ELOVL1^+/-^ (Chi^2^ *P* = ns) *Abcd1*^*y/*-^;*Cnp-ELOVL1*^*+/-*^ males display normal development, including normal body weight compared to control mice and are fertile.

**Fig 3:**
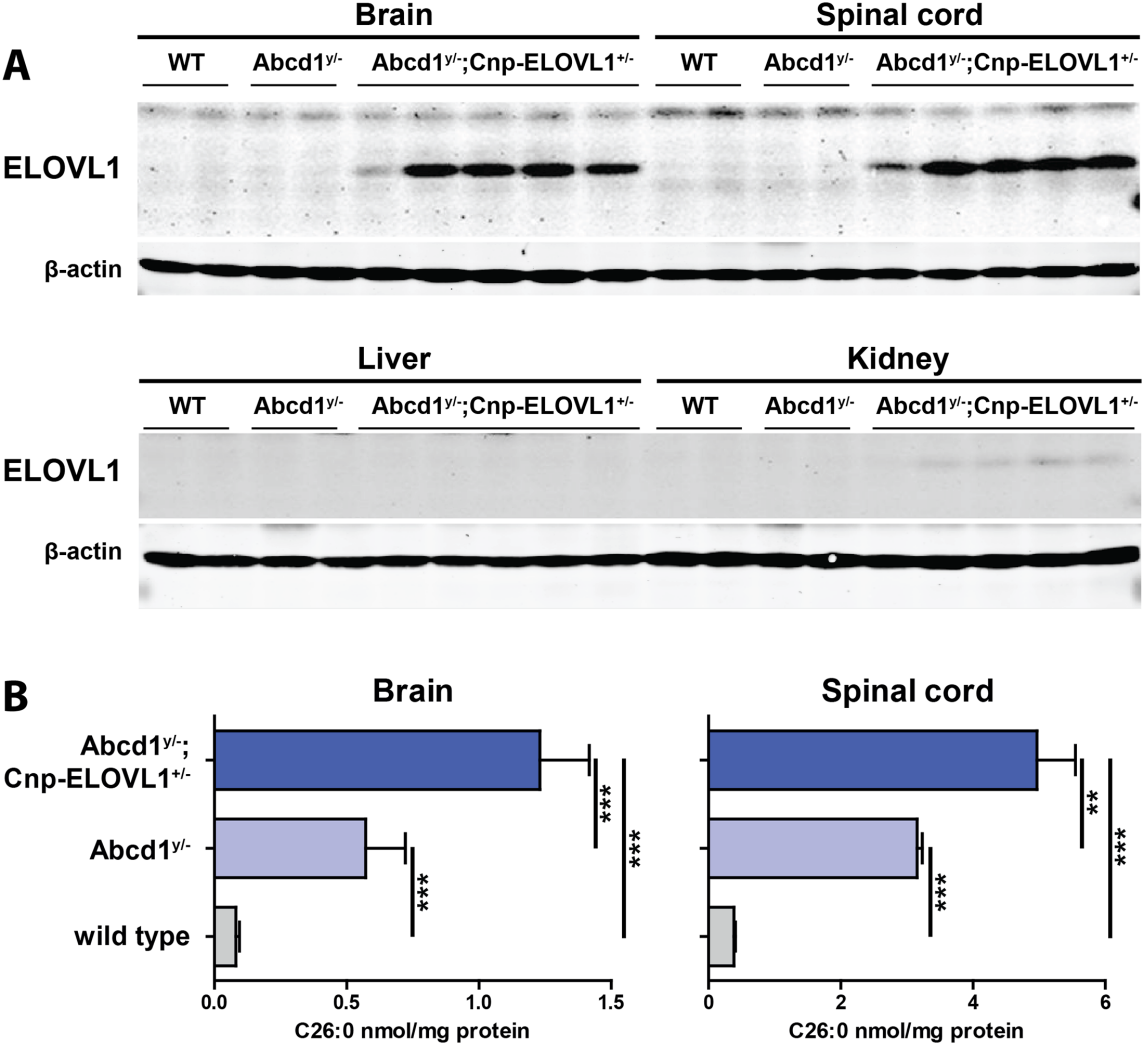
Generation and characterization of *Abcd1*;*Cnp-ELOVL1* mice. **(A)** ELOVL1 protein expression in brain and spinal cord from wild type, *Abcd1*^*y/-*^ knockout and *Abcd1*^*y/*-^;*Cnp-ELOVL1*^*+/-*^. **(B)** C26:0 levels in brain and spinal cord from wild type (n = 6), *Abcd1*^*y/-*^ knockout (n = 6) and *Abcd1*^*y/*-^;*Cnp-ELOVL1*^*+/-*^ (n = 6) mice. Data are mean ± SD. ***p<0.001 by unpaired student’s t-test.

To assess the biochemical effect of oligodendrocyte-specific ELOVL1 over-expression in *Abcd1* mice, ELOVL1 protein and VLCFA levels were measured in 8 month old wild type, *Abcd1*^*y/-*^ knockouts and *Abcd1*^*y/*-^;*Cnp-ELOVL1*^*+/-*^ mice. Western blot analysis of total brain, spinal cord, liver and kidney demonstrated a strong expression of transgenic ELOVL1 protein in the CNS of *Abcd1*^*y/*-^;*Cnp-ELOVL1*^*+/-*^ mice, no expression in liver and a faint signal in kidney (Fig 3A). This confirmed CNS specific ELOVL1 modulation.

Total VLCFA levels were measured in brain and spinal cord homogenates generated from wild type, *Abcd1*^*y/-*^ and *Abcd1*^*y/*-^;*Cnp-ELOVL1*^*+/-*^ mice (Fig 3B). Compared to wild type mice, in *Abcd1* knockouts C26:0 levels were increased 7-fold in brain and 8-fold in spinal cord. In *Abcd1*^*y/*-^;*Cnp-ELOVL1*^*+/-*^ mice C26:0 levels were increased 15-fold in brain and 12-fold in spinal cord, respectively.

Interestingly, the analysis of C26:0 levels in whole brain homogenates from *Abcd1* knockouts with ubiquitous ELOVL1 over-expression (*Abcd1*^*y/*-^;*CMV-ELOVL1*^*+/-*^) and *Abcd1* knockout with ELOVL1 overexpression restricted to oligodendrocytes (*Abcd1*^*y/*-^;*Cnp-ELOVL1*^*+/-*^) revealed similar C26:0 levels (20-fold and 15-fold, respectively). This indicates that oligodendrocytes are largely responsible for the total VLCFA synthesis in brain. These data are in agreement with earlier work that reported similar C26:0 levels in whole brain lysates and in purified myelin derived from oligodendrocyte-specific peroxisome knockout mice [36]. Taken together, these data strongly indicate that oligodendrocytes are largely responsible for the total VLCFA degradation and VLCFA synthesis activity of the brain.

### C26:0-lysoPC Is Highly Elevated in Central Nervous Tissue

**Fig 4:**
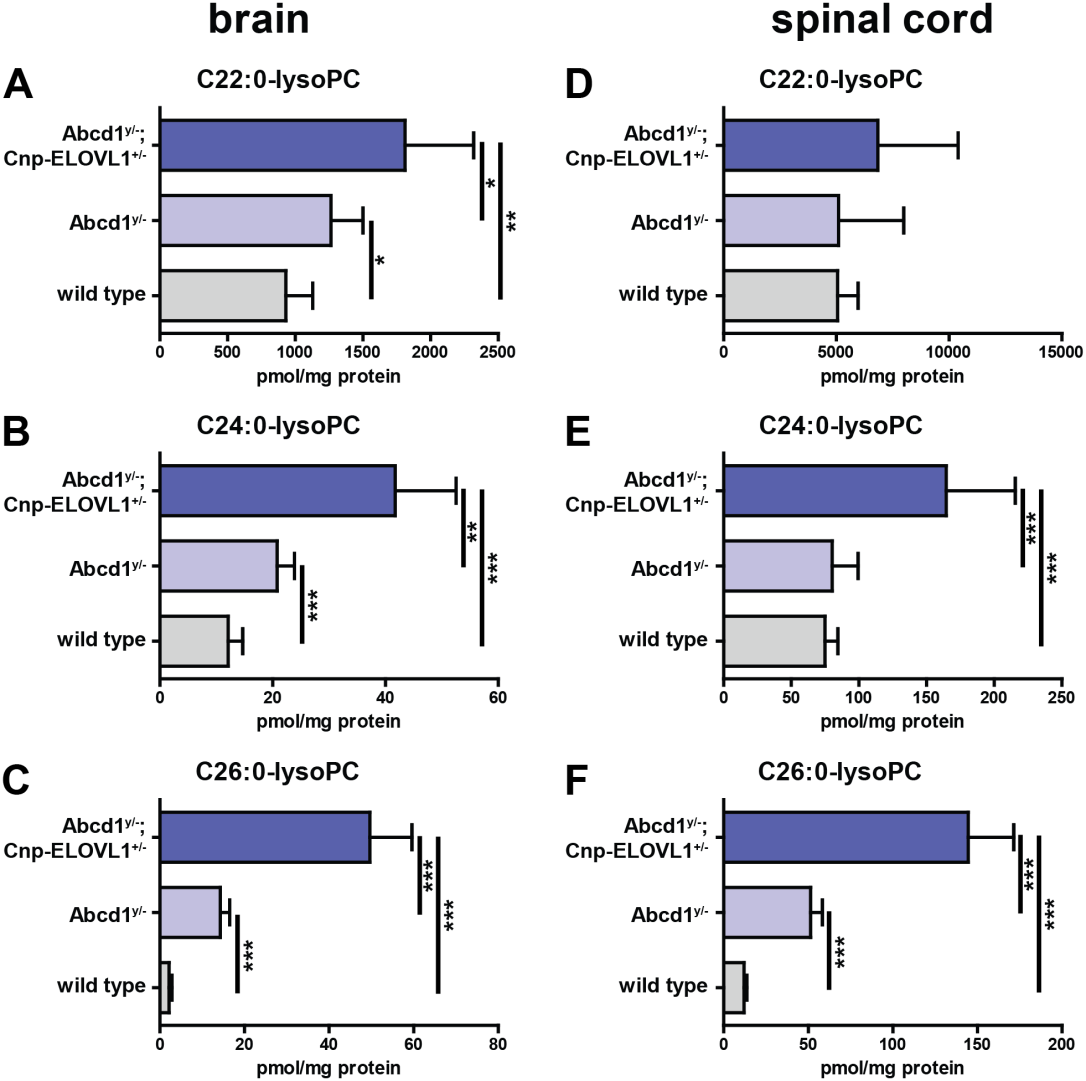
C26:0-lysoPC is highly elevated in central nervous tissue. C22:0-lysoPC, C24:0-lysoPC and C26:0-lysoPC levels in brain and spinal cord from wild type (n = 6), *Abcd1*^*y/-*^ knockout (n = 6) and *Abcd1*^*y/*-^;*Cnp-ELOVL1*^*+/-*^ (n = 6) mice. Data are mean ± SD. *p<0.05, **p<0.01, ***p<0.001 by unpaired student’s t-test.

The currently available biomarkers for ALD are excellent for establishing the diagnosis (for instance, total C26:0 in plasma or blood cells), but do not correlate with, phenotype, disease severity or pattern of progression. In clinical trials C26:0 is therefore a biomarker of doubtful significance, which implies that new biomarkers are urgently needed. In recent years, it has been demonstrated that 1-hexacosanoyl-sn-glycero-3-phosphocholine (C26:0-lysoPC) is already elevated at birth in blood of newborns with ALD by 5-fold [13–15]. We investigated whether C26:0-lysoPC levels are directly associated with elevated total C26:0 levels observed in our mouse models and measured C26:0-lysoPC in mouse brain and spinal cord. Analysis of brain and spinal cord samples from wild type, *Abcd1*^*y/-*^ knockout and *Abcd1*^*y/*-^;*Cnp-ELOVL1*^*+/-*^ mice revealed that C26:0-lysoPC is highly elevated in *Abcd1*^*y/-*^ knockouts and *Abcd1*^*y/*-^;*Cnp-ELOVL1*^*+/-*^ mice (Fig 4). Compared to wild type mice, in *Abcd1* knockouts C26:0-lysoPC levels were increased 4-fold in brain and 6-fold in spinal cord, and in *Abcd1*^*y/*-^;*Cnp-ELOVL1*^*+/-*^ C26:0-lysoPC levels were increased 13-fold in brain and 24-fold in spinal cord.

### C26:0-Carnitine Is Highly Elevated in Central Nervous Tissue

**Fig 5:**
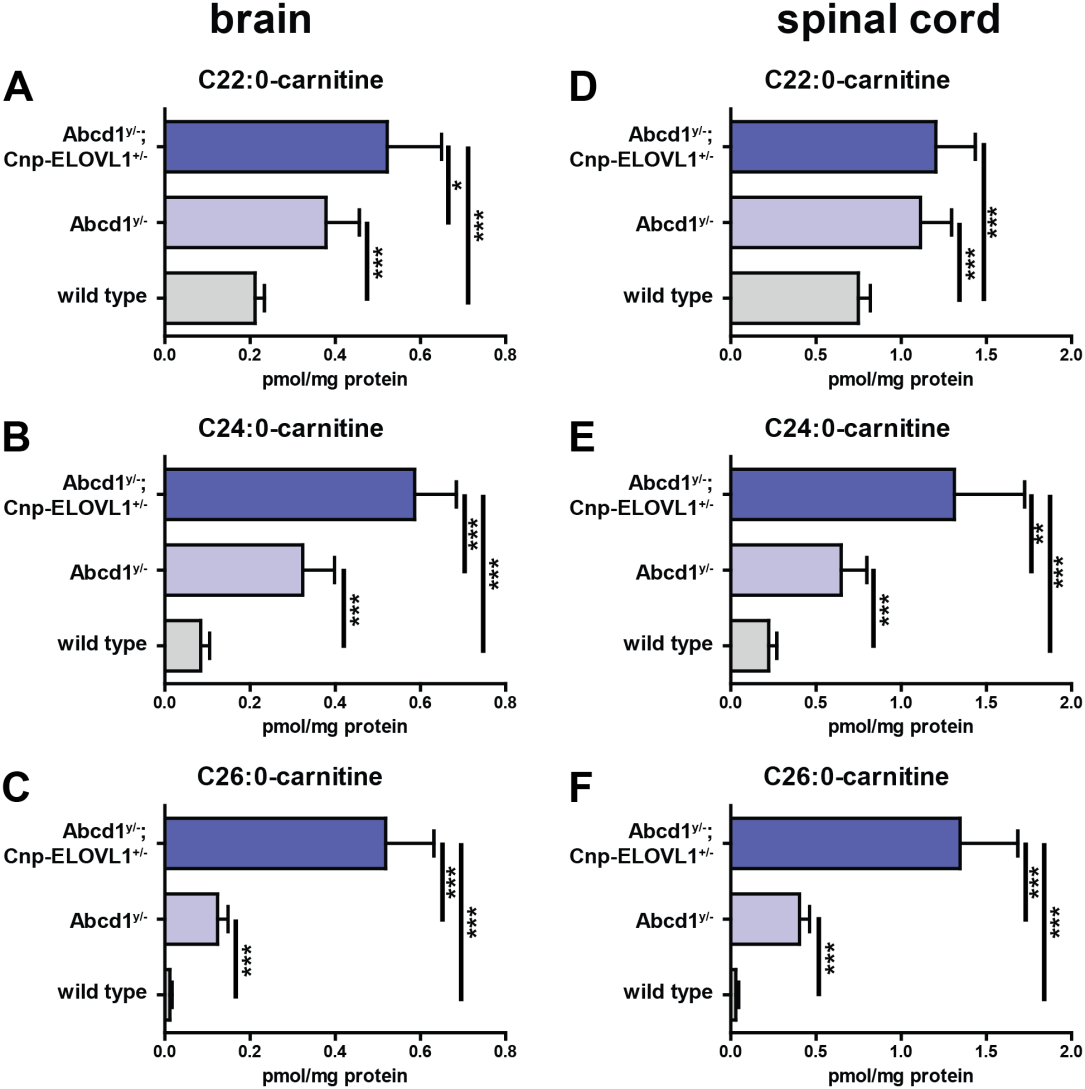
C26:0-carnitine is highly elevated in central nervous tissue. C22:0-carnitine, C24:0-carnitine and C26:0-carnitine levels in brain and spinal cord from wild type (n = 6), *Abcd1*^*y/-*^ knockout (n = 6) and *Abcd1*^*y/*-^;*Cnp-ELOVL1*^*+/-*^ (n = 6) mice. Data are mean ± SD. **p<0.01, ***p<0.001 by unpaired student’s t-test.

In 2003, increased C24:0-carnitine and C26:0-carnitine levels were reported in plasma and bloodspots from patients with either a peroxisomal biogenesis disorder or D-bifunctional protein deficiency [37]. No increase was reported in plasma from ALD patients. Importantly, bloodspots from ALD patients were not included in the study. Significantly elevated VLCFA acylcarnitines were also reported in urine samples from patients with a peroxisomal biogenesis disorder [38]. This latter study also did not include samples from ALD patients. We investigated whether VLCFA-acylcarnitines are also elevated in our ALD mouse models. Analysis of brain and spinal cord samples from wild type, *Abcd1*^*y/-*^ knockout and *Abcd1*^*y/*-^;*Cnp-ELOVL1*^*+/-*^ mice revealed that C26:0-carnitine levels are highly elevated in *Abcd1*^*y/-*^ knockouts and *Abcd1*^*y/*-^;*Cnp-ELOVL1*^*+/-*^ mice (Fig 5). Compared to wild type mice, in *Abcd1*^*y/-*^ knockouts C26:0-carnitine levels were increased 10-fold in brain and 9-fold in spinal cord, and in *Abcd1*^*y/*-^;*Cnp-ELOVL1*^*+/-*^ C26:0-carnitine levels were increased 40-fold in brain and 33-fold in spinal cord.

### Comparison of C26:0, C26:0-lysoPC and C26:0-carnitine in Mouse and Human Blood

In the USA, New York State has initiated ALD newborn screening in 2014 and in Europe the Netherlands will start ALD newborn screening in the near future. The current method for diagnosing ALD is sensitive and specific, but it involves a dedicated and separate analysis for C26:0-lysoPC. In the search for new biomarkers for ALD that are more sensitive than total C26:0 and easier to measure than C26:0-lysoPC we compared C26:0 levels with C26:0-lysoPC and C26:0-carnitine in mouse and then expanded this analysis to human plasma and bloodspots.

**Fig 6:**
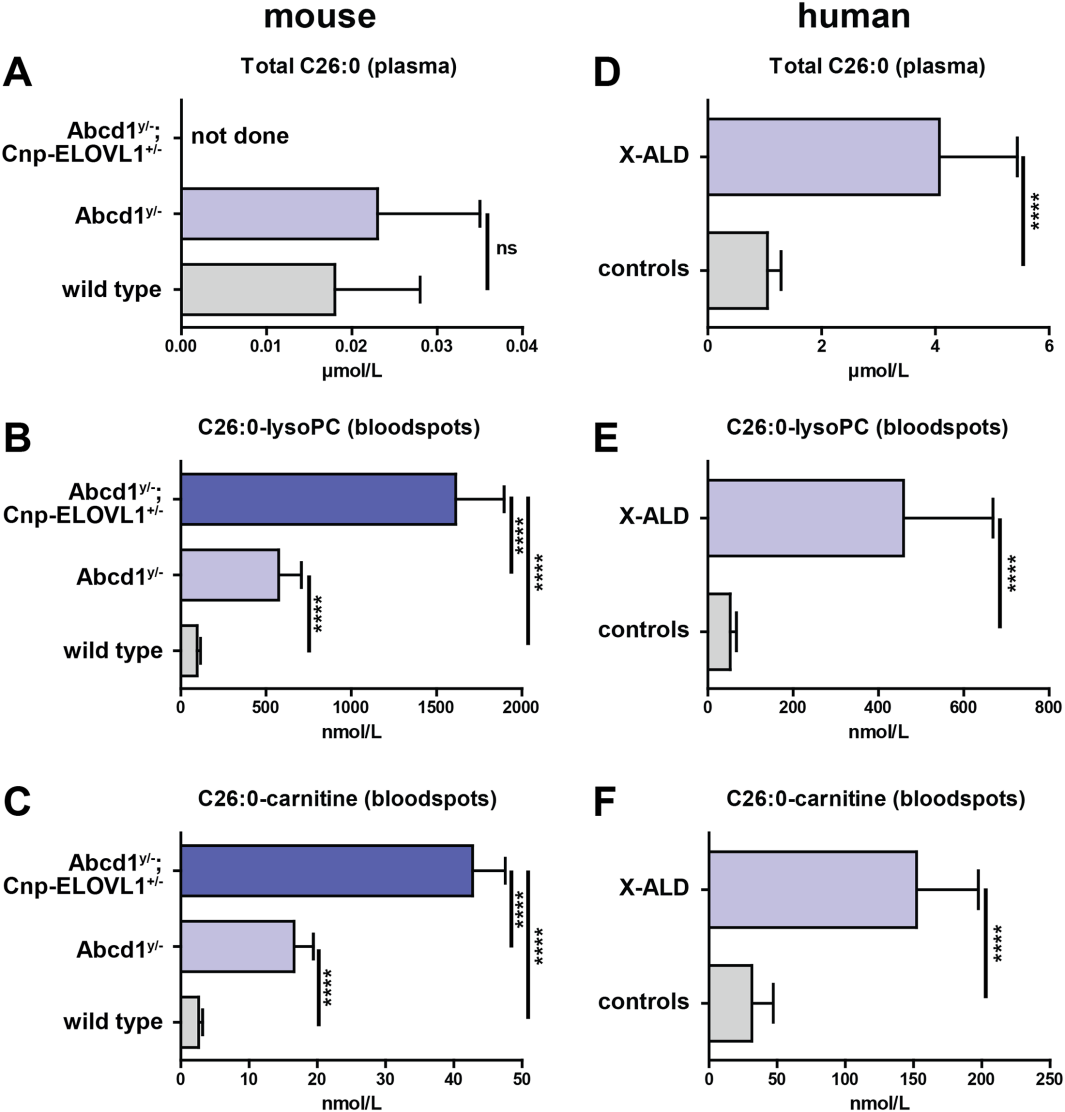
C26:0-carnitine is highly elevated in mouse and human bloodspots. **(A)** plasma total C26:0 in wild type (n = 8), *Abcd1*^*y/-*^ knockout (n = 10) and *Abcd1*^*y/*-^;*Cnp-ELOVL1*^*+/-*^ (n = 6) mice. **(B)** bloodspot C26:0-lysoPC in wild type (n = 8), *Abcd1*^*y/-*^ knockout (n = 10) and *Abcd1*^*y/*-^;*Cnp-ELOVL1*^*+/-*^ (n = 6) mice. **(C)** bloodspot C26:0-carnitine in wild type (n = 8), *Abcd1*^*y/-*^ knockout (n = 10) and *Abcd1*^*y/*-^;*Cnp-ELOVL1*^*+/-*^ (n = 6) mice. **(D)** plasma total C26:0 in controls (n = 23) and ALD patients (n = 10). **(E)** bloodspot C26:0-lysoPC in controls (n = 23) and ALD patients (n = 10). **(F)** bloodspot C26:0-carnitine in controls (n = 23) and ALD patients (n = 10). Data are mean ± SD. ****p<0.0001 by unpaired student’s t-test.

In agreement with earlier reports total plasma C26:0 is not increased in *Abcd1*^*y/-*^ knockouts when compared to wild type mice (Fig 6A)[39]. Analysis of dried bloodspots, however, revealed that C26:0-lysoPC was increased 6-fold in *Abcd1*^*y/-*^ knockouts and 17-fold in *Abcd1*^*y/*-^;*Cnp-ELOVL1*^*+/-*^ mice (Fig 6B). Furthermore, C26:0-carnitine was increased 6-fold in *Abcd1*^*y/-*^ knockouts and 16-fold in *Abcd1*^*y/*-^;*Cnp-ELOVL1*^*+/-*^ mice (Fig 6C). When compared to age-related adult controls, in ALD patients plasma total C26:0 was increased 4-fold (Fig 6D), while in bloodspots C26:0-lysoPC was increased 9-fold (Fig 6E), and C26:0-carnitine was increased 5-fold (Fig 6F).

Previously, it was demonstrated that brain VLCFA levels correlate with the clinical phenotype [20]. Biochemical analysis of normal-appearing grey and white matter that was dissected from frontal, parietal or occipital lobes from 17 ALD patients and 19 age-matched controls revealed that in comparison with age-matched controls, C26:0 levels were increased 3-fold in cerebral ALD patients, and 1.9-fold in AMN patients. These results suggest that the accumulation in brain VLCFA levels precedes histopathological alterations and are an important factor in the development of cerebral disease. Our data demonstrate that, at least in mice, total plasma C26:0 levels do not correlate with C26:0 levels present in either spinal cord or brain. Interestingly, both C26:0-lysoPC and C26:0-carnitine levels in mouse bloodspots correlate with the C26:0, C26:0-lysoPC and C26:0-carnitine levels found in brain and spinal cord. We conclude that C26:0-lysoPC and C26:0-carnitine are new biochemical biomarkers for ALD that reflect the VLCFA levels present in the central nervous system. Further studies are warranted to investigate whether bloodspot C26:0-lysoPC and/or C26:0-carnitine levels correlate with disease severity in ALD patients.

## Conclusions

We anticipated that increased VLCFA levels in the central nervous system of *Abcd1* knockout mice play a role in the clinical phenotype of the *Abcd1* knockout mouse and may lead to the identification of new biomarkers. These are urgently needed as C26:0 levels do not correlate with disease severity. To this end, we developed an *Abcd1* knockout mouse with a Cre-inducible *ELOVL1* transgene. Our data demonstrate that enhanced in vivo expression of ELOVL1 results in increased VLCFA synthesis and consequently increased VLCFA levels. Transgenic mice displayed highly elevated levels of C26:0-lysoPC in central nervous tissue. Furthermore, we identified a new biomarker, C26:0-carnitine, in brain and spinal cord tissue from *Abcd1;Cnp-ELOVL1* mice. Interestingly, highly elevated levels of C26:0-carnitine were also demonstrable in easily accessible material, i.e. bloodspots of both transgenic mice and ALD patients with the AMN phenotype. Our finding that C26:0-carnitine is elevated in dried bloodspots from males with ALD could, after validation of these results in a newborn bloodspots, result in a simple addition of C26:0-carnitine to the often already existing high throughput screen for mitochondrial fatty acid disorders thereby eliminating the need for a separate C26:0-lysoPC analysis. We anticipate that this may lead to a faster inclusion of ALD in newborn screening programs in countries that already screen for other inborn errors of metabolism.
